# Are we ready for "green surgery" to promote environmental sustainability in the operating room? Results from the WSES STAR investigation

**DOI:** 10.1186/s13017-024-00533-y

**Published:** 2024-01-24

**Authors:** Francesca Dal Mas, Lorenzo Cobianchi, Daniele Piccolo, Jeremy Balch, Helena Biancuzzi, Walter L. Biffl, Stefano Campostrini, Enrico Cicuttin, Federico Coccolini, Dimitris Damaskos, Amanda C. Filiberto, Claudia Filisetti, Gustavo Fraga, Simone Frassini, Paola Fugazzola, Timothy Hardcastle, Haytham M. Kaafarani, Yoran Kluger, Maurizio Massaro, Jacopo Martellucci, Ernest Moore, Federico Ruta, Massimo Sartelli, Philip F. Stahel, George Velmahos, Dieter G. Weber, Fausto Catena, Tyler J. Loftus, Luca Ansaloni, the STAR Study Group

**Affiliations:** 1 Collegium Medicum, University of Social Sciences, Łodz, Poland; 2 Department of Management, Ca’ Foscari University, Venice, Italy; 3 Department of Clinical, Surgical, Diagnostic and Pediatric Sciences, University of Pavia, Via Alessandro Brambilla, 74, 27100 Pavia, PV Italy; 4 General Surgery, Department, IRCCS Policlinico San Matteo Foundation, Pavia, Italy; 5 Unit of Neurosurgery, Department of Head-Neck and Neuroscience, ASUFC Santa Maria Della Misericordia, Udine, Italy; 6 Department of Surgery, University of Florida Health, Gainesville, FL USA; 7 Department of Economics, Ca’ Foscari University, Venice, Italy; 8 Division of Trauma and Acute Care Surgery, Scripps Memorial Hospital La Jolla, La Jolla, CA USA; 9 General, Emergency and Trauma Surgery Dept, Pisa University Hospital Pisa, Pisa, Italy; 10 General and Emergency Surgery, Royal Infirmary of Edinburgh, Edinburgh, UK; 11 Department of Pediatric Surgery, Buzzi Children’s Hospital, Milan, Italy; 12 Division of Trauma Surgery, Department of Surgery, School of Medical Sciences, University of Campinas (Unicamp), Campinas, SP Brazil; 13 Department of Surgical Sciences, Nelson R Mandela School of Clinical Medicine, University of KwaZulu-Natal, Durban, 4001 South Africa; 14 Trauma and Burns Services, Inkosi Albert Luthuli Central Hospital, Mayville, 4058 South Africa; 15 Harvard Medical School, Boston, MA USA; 16 Division of Trauma, Emergency Surgery, and Surgical Critical Care, Massachusetts General Hospital, Boston, MA USA; 17 Division of General Surgery, Rambam Health Care Campus, Haifa, Israel; 18 Department of Surgery, Careggi University Hospital, Florence, Italy; 19 Ernest E Moore Shock Trauma Center at Denver Health, Denver, CO USA; 20 General Direction, ASL BAT (Health Agency), Andria, Italy; 21 Department of Surgery, Macerata Hospital, Macerata, Italy; 22 Department of Surgery, Brody School of Medicine, East Carolina University, Greenville, NC USA; 23 Department of General Surgery, Royal Perth Hospital, University of Western Australia, Perth, Australia; 24 Acute Care Surgery Unit, Department of Surgery and Trauma, Maurizio Bufalini Hospital, Cesena, Italy

**Keywords:** Environmental Sustainability, Sustainability, Trauma and emergency surgery; survey; Green

## Abstract

**Background:**

The importance of environmental sustainability is acknowledged in all sectors, including healthcare. To meet the United Nations Sustainable Development Goals 2030 Agenda, healthcare will need a paradigm shift toward more environmentally sustainable practices that will also impact clinical decision-making. The study investigates trauma and emergency surgeons’ perception, acceptance, and employment of environmentally friendly habits.

**Methods:**

An online survey based on the most recent literature regarding environmental sustainability in healthcare and surgery was created by a multidisciplinary committee and endorsed by the World Society of Emergency Surgery (WSES). The survey was advertised to the 917 WSES members through the society’s website and Twitter/X profile.

**Results:**

450 surgeons from 55 countries participated in the survey. Results underline both a generally positive attitude toward environmental sustainability but also a lack of knowledge about several concepts and practices, especially concerning the potential contribution to patient care.

**Discussion:**

The topic of environmental sustainability in healthcare and surgery is still in its infancy. There is a clear lack of salient guidance and knowledge, and there is a critical need for governments, institutions, health agencies, and scientific societies to promote, disseminate, and report environmentally friendly initiatives and their potential impacts while employing an interdisciplinary approach.

**Supplementary Information:**

The online version contains supplementary material available at 10.1186/s13017-024-00533-y.

## Background

The *World Commission on Environment and Development (WCED*), established in 1983 under the United Nations (UN), published its final report entitled "*Our Common Future in 1987.*" This report is still considered a cornerstone of environmental issues and their connection to global socio-economic imbalances [1]. This document established the commonly held definition of sustainable development, which meets the needs of the present without compromising those of the future. This definition has been referred to by subsequent global documents and conferences until the adoption in September 2015 of the United Nations 2030 Agenda for Sustainable Development [2]. With its 17 *Sustainable Development Goals (SDGs*) and 169 *targets*, the 2030 Agenda offers a new vision of integrating the three dimensions of sustainability: environmental, social, and economic. The environmental dimension is integrated throughout the text, with a full convergence of social sustainability goals, poverty eradication [3], and environmental protection.

Sustainability, then, continues to be increasingly imperative, as dominance over nature and its resources has given way to a concrete need to achieve greater environmental awareness and balance [1]. This need, evident in all three dimensions of sustainability [4], has also resonated in healthcare, where we define sustainability as the delivery of high-quality healthcare today without jeopardizing the same quality of care for future generations. Most global efforts on healthcare sustainability, however, have so far referred to the social aspects of care, including universal healthcare, ensuring inclusion, and reducing disparities in access and quality of care [5, 6]. Economic sustainability has also been the focus of debate in the literature due to frequent budget cuts, especially in high-income countries characterized by an aging population with chronic diseases and the consequent need for more health services [7]. Instead, environmental sustainability has often remained in the background due to the priority given to social and economic aspects in access to care. However, ecological factors must be accounted for as a priority, if we think, for example, at the case of gallium, gadolinium, and germanium [8].

According to the World Health Organization (WHO), health is a state of complete physical, mental, and social well-being and not simply the absence of disease or infirmity [9]. In this context, healthcare providers are the main actors in promoting good health. Access to healthcare and surgical care is strongly influenced by many factors, such as economic conditions or national health policies, which vary greatly among countries, populations, and individuals [3, 5]. Regardless of the system being considered, hospitals and healthcare institutions represent the Health Care System as a whole, enabling the pursuit of medical, surgical, and nursing care for sick or injured individuals. However, recent studies have unveiled how traditional hospital facilities may paradoxically instead have an indirect negative impact on public health and, more broadly, on the community and environment in which they are embedded [10]. The health sector, in fact, has a significant impact within a global context of climate change, air pollution, and increasing waste. The healthcare business employs millions of people, caters to many stakeholders, manages multiple facilities, produces investments and consumable goods and services, and engages with extensive supply chains involving both the public and private sectors in all countries. The healthcare sector represents, therefore, a field of high environmental impact, and it is necessary to devise strategies aimed at balancing performance, quality, and sustainability [11, 12]. Such strategies must go beyond the construction of sustainable buildings, defined in the international literature as "healthy," [13] and must, therefore, also focus on waste and energy generation and management [14]. Although there are no global statistics, a study by the UK National Healthcare System (NHS) showed that healthcare contributes to the creation of 4.6% of the UK's absolute carbon emissions and about 25% in terms of public sector emissions [15]. Awareness of environmental impact allows for targeted strategies for containing and reducing that impact [14, 16].

International literature and practice are thus devoting increasing attention to the issue of environmental sustainability in healthcare and surgery. The NHS and the UK medical and surgical colleges are forerunners in an area where specific literature is currently scarce [17]. These institutions have begun a series of studies and pieces of research aimed at measuring the environmental impact of the entire system and identifying shared strategies to be implemented in the short, medium, and long term [17, 18]. The UK analysis dictates guidelines from a structural perspective on the modernization and energy efficiency of buildings and management processes (especially in terms of procurement), but they are not sufficient. True change needs to happen at every level, and it is crucial to involve and raise awareness among healthcare professionals, who should consider the environmental impact of their clinical decisions. To address this problem, in May 2022, the English *Royal College of Surgeons of England* published the "*Sustainability in the operating theatre: Guide to good practice,*" [19] which promotes suggestions to reduce solid waste, stimulate lower-impact purchasing and water conservation, reduce patient travel through telemedicine, and, in general terms, support a cultural shift toward lower-impact clinical and surgical practices. In November 2022, the *Royal College of Surgeons of* Edinburgh, the *Royal College of Surgeons* of England and the *Royal College of Physicians and Surgeons* of Glasgow published the "*Intercollegiate Green Theatre Checklist Compendium of Evidence,*" [20] a 16-item checklist covering the entire surgical journey, from anesthesia to operative preparation, intraoperative equipment, and postoperative care. According to the UK professional bodies, surgical activity is particularly carbon-intensive [21]: in fact, it is estimated that a single operation generates between 150 and 170 kg of CO_2_e, equivalent to driving 720 km in an average gasoline car.

Recent studies and contributions by the NHS and the UK professional organizations are fueling discussion on environmental sustainability within healthcare, medicine, and surgery. This topic is emerging as one of the most promising avenues for healthcare research, practice, and policy in the upcoming years. The first step is to understand environmental practices, both in clinical processes and in the management of buildings and facilities, particularly hospitals, alongside the perceptions and attitudes of health professionals (whether managers, clinicians, technicians, or administrators) toward change management and environmental practices directly related to their daily tasks and activities.

Building on these premises, the present study aims to investigate this emerging issue by understanding environmental practices in hospitals and healthcare institutions and the propensity and degree of acceptance by clinicians toward the importance and adoption of policies aimed at environmental sustainability. Specifically, this STAR study (**S**ustainability in **T**rauma and emergency surgery **A**cceptance **R**ate) focused on the perception of environmental sustainability and change management by surgeons and physicians belonging to the World Society of Emergency Surgery (WSES) to understand its degree of awareness and maturity. Moreover, the survey had the goal to map the presence or absence of specific sustainability actions and practices in conducting clinical activities.

## Methods

### Design and setting

Through a population-based online questionnaire, this study gathered demographic, knowledge-based, and practice-based information regarding sustainability practices and acceptance dynamics among surgeons and physicians belonging to the WSES community. The online questionnaire was created in English using Google Forms [22, 23]. The Checklist for Reporting Results of Internet E-Surveys (CHERRIES) [24] was followed.

The first draft of the questionnaire was designed by one general surgery expert (LC) and one management expert (FD), in accordance with the most recent literature, specifically, the recent documents published by the Royal Colleges of the United Kingdom, the literature on stakeholder engagement [25], knowledge translation [26], and sustainability in surgery [21, 27]. Descriptive statistics were gathered following previous studies [22, 23, 28, 29].

The first draft was submitted in advance for analysis and review by twenty-six experts: eighteen in the emergency surgery field, appointed within the WSES, three in business management and organization, one in social statistics, one in administrative law, and three in STEM fields.

Before the initiative's official launch, the online survey was completed by a sample of surgeons and healthcare managers as a pilot test to prevent errors.

The questionnaire was accessible from mid-February 2023 until mid-June 2023. All 917 WSES members were invited to participate in the survey via email. Additionally, the initiative was shared on the organization's website and Twitter account. In addition, email invitations were sent to the members of the Team Dynamics Study Group [22, 23, 28, 29]. Three email messages were sent as reminders. WSES membership was not required to participate in the survey. Nevertheless, it is assumed that the overwhelming majority of participants are drawn from the 917 WSES members to whom the research initiative was communicated, yielding 450 fully filled questionnaires, with a response rate of 49%.

The invitation email contained extensive information on the initiative's objectives and rationale, the anticipated time frame (approximately 10 min), and the possibility of joining the STAR Study Group to conduct additional research and disseminate the results. The participants' identities were kept concealed unless they wished to join the study group. The researchers' identities and the research protocol were kept confidential. Early findings were presented and discussed during the International WSES congress held in Pisa, Italy, on 20–23 June 2023.

### Survey

The first group of questions aimed at understanding the participants’ features. Questions were derived from previous investigations into Team Dynamics [22, 23, 28, 29]. Surgeons were asked to disclose their gender, years of experience in trauma/emergency surgery, type of institution (academics or community), country of residence and work, role within the institution, possible participation within a trauma team (institutionalized or not, and of which type), background of the possible trauma leader, educational modules/courses attended, and presence of diverse members within the team.

The second group of questions aimed at understanding the perception and the concept of green and environmental sustainability applied to surgery through a yes/no question and an open one, in which medical doctors had to describe the term.

The third section was about sustainability acceptance and change management. Surgeons and physicians had to think about their clinical practice and, on a 5-point Likert scale, rate twenty-six statements on various topics, from stakeholders to academic research, from procurement to reporting.

The fourth section was concerned with environmental sustainability habits in surgical practice. Participants had to rate twenty-three patterns on a 5-point Likert scale, mainly gathered from the UK professional bodies’ lists and reports. Moreover, they had to evaluate an additional nineteen items concerning hospital policies, operative supplies, and waste management protocols. They also had to select the most appropriate means of translation and communication of sustainability practices. Last but not least, participants were required to rate some general benefits of engaging in green habits or tasks.

The survey’s questions related to Sects. "Methods", "Results", and "Discussion" are reported in Appendix 1.

### Statistical analysis

Manual coding was employed concerning the qualitative question about the understanding of green/environmental sustainability applied to surgery. The given responses were manually coded by two researchers (LC and FDM), who rated each statement as concordant, discordant, or inconclusive, following the analysis of Woltz et al. [30] and Cobianchi et al. [22, 29].

The association between categorical variables was assessed using Pearson’s Chi-squared test. For the distribution of ordinal or continuous variables across dual independent groups, the Wilcoxon rank sum test was implemented. In cases involving multiple group comparisons, the Kruskal–Wallis rank sum test was employed. A p value threshold of 0.05 was set as the benchmark for statistical significance. All p values cited are two-tailed. All analyses were executed utilizing R software (RStudio version 2023.06.1.524).

## Results

### Participants

Four hundred and fifty surgeons and physicians responded to the questionnaire.

The sample consisted of 89 female surgeons (20%), 356 male surgeons (79%), and five individuals who wished to remain anonymous. The majority of participants (339, or 75% of the sample) were from academic institutions. Although a diversity of roles was indicated, the majority of surgeons (180, or 40%) were senior consultants. Seventy four (16%) of the total sample were department directors. The majority of participants (385, or 86%) were members of a formalized emergency surgery team. The surgeons' years of experience in the discipline ranged from 1 to 36, with a mean of 15 years and a standard deviation of 9. Participants represented 55 different countries. However, the sample was not evenly distributed, with the majority of surgeons working in Italy (145, or 32,22%). In particular, 322 respondents (72%) were from the ten countries with the most aggregate participants.

Table 1 provides descriptive statistics regarding the individuals and institutions that participated in the study.

**Table 1:** Descriptive statistics about participants

Item	Number of responses	% of total responses
*Participants*	*450*	*100.00*
Males	356	79.11
Females	89	19.78
Prefer not to answer	5	1.11
*Kind of Institution*	*450*	*100.00*
Academic	339	75.33
Non-academic	111	24.67
*Role/position*	*450*	*100.00*
Senior Consultant	180	40.00
Board-certified surgeon	123	27.33
Division Chief or Head	74	16.44
Resident	73	16.22
*Part of an emergency surgery team*	*450*	*100.00*
Yes	385	85.56
No	65	14.44
*Number of represented countries*	*55*	
*Ten most represented countries*		
Italy	145	32.22
Greece	51	11.33
United Kingdom	36	8.00
Spain	23	5.11
Malaysia	17	3.78
Turkey	15	3.33
United States	11	2.44
Brazil	8	1.78
Bulgaria	8	1.78
Romania	8	1.78
*Participants from the ten most represented countries*	*322*	*71.56*
	*Mean*	*Standard deviation*
Years of experience	14.82	9.4
Minimum	1	
Maximum	36	

### Perception of environmental sustainability

Regarding the perception and understanding of the concept of green/environmental sustainability, participants were first asked if they were familiar with the term, using a “yes or no” question. Three hundred and six (68%) replied they were, and the remaining 144 (32%) declared they were not. Moreover, participants were asked to describe the meaning of green/environmental sustainability applied to surgery. As specified and in line with previous studies [28–30], two principal investigators rated each given statement as concordant, discordant, or inconclusive.

To be rated as concordant, definitions needed to stress at least some of the concepts, such as a wise use of resources, the reduction in carbon footprint, energy savings, or smart waste management. Interestingly, less than half of the participants (182, 40% of the sample) provided definitions that could be rated as concordant according to the abovementioned criterion. 129 participants (29% of the sample) were rated as inconclusive as they gave responses that were incomplete, showing only a partial view of the phenomenon. The remaining 139 surgeons (31%) gave answers that did not fit the general definition of environmental sustainability.

The following Table 2 reports some examples of answers that were rated as concordant, inconclusive, and discordant.

**Table 2:** Examples and ways of rating the given answers to the question: How would you describe green/environmental sustainability applied to surgery?

Rated as	Given answer	Reason for rating
Concordant	“Any activities applied in surgery that will limit/reduce the carbon footprint and waste with an environmentally friendly approach” [response #1]	The definition recalls the need to limit or reduce carbon footprint
	“Environmentally-aware surgical activity, decreasing costs and decreasing resource expenditure, including through the use of sustainable practices” [response #9]	The definition recalls the concept of resource awareness
	“Working in a way that does not damage the future generations' resources” [response #39]	The definition recalls the need to preserve resources for future generations
	“To understand the impact and reduce the amount of waste produced by the healthcare facilities and activities” [response #54]	The definition recalls the need to reduce the impact of waste
	“Minimizing carbon footprint, maximizing recycling and reusing products “ [response #116]	The definition recalls the need to limit or reduce carbon footprint
	“Optimizing resources without compromising care” [response #150]	The definition recalls the need to preserve resources for future generations
	“Going green means using environmentally friendly products and services. Sustainability means using products or services in a way that does not damage the future generations' resources. Hence, while a final product may be green, its manufacturing or production process may not be sustainable at all” [response #186]	The definition recalls the need to preserve resources for future generations
	“The principles that can be applied to surgery which have a positive impact on the environment such as reducing waste, minimizing energy use, using environmental-friendly products, reducing transport emission” [response #199]	The definition recalls the need to preserve resources
	“Promoting low-carbon alternative products and processes in surgery” [response #299]	The definition recalls the need to limit or reduce carbon footprint
Inconclusive	“I don’t know” [response #40]	Unclear in its meaning
	“Very important for surgery” [response #90]	
	“The balance between man, animal, and human should be protected… “[response #174]	
	“Future” [response #347]	
	“Applicable, but there is a lack of information on this subject “ [response #415]	
Discordant	“Cost effective value-based medicine” [response #131]	Costs are relevant but refer to a different dimension of sustainability
	“Awareness of operating room costs” [response #260]	
	“Culture” [response #339]	Culture may matter, but it is central
	“It is not a priority. Safety of the patient is first goal” [response #405]	The concept of patient safety is different than the requested one

### Sustainability acceptance and change management

Concerning the first group of general items to measure acceptance, participants gave the best evaluation to general statements, such as the need for Governments to incentivize green surgical practices or the global importance of sustainability principles in all economic fields. They also focused on surgical instruments and on the requirement to reduce their environmental impacts. They were also optimistic about the potential contribution of technology to greener practices.

The items with a less favorable evaluation concerned the role of patients in appreciating and trusting greener surgical practices.

Table 3 reports the results of the item belonging to the first question.

**Table 3:** Results—Sustainability acceptance and change management

Think of your clinical practice. On a scale from 1 to 5, where 1 = strongly disagree and 5 = strongly agree, how would you rate the following statements?		
	MEAN	SD
[Governments should incentivize green surgical practices]	4.30	0.89
[Surgical instruments should consider environmental efforts (eg. limiting the use of energy, non-recyclable materials and the creation of waste)]	4.21	0.87
[Technology can lead to more sustainable surgical practices]	4.21	0.88
[Green management should be a priority in all the industrial sectors]	4.19	0.92
[Clinicians should be educated about environmental issues and outcomes connected with clinical activities and choices]	4.17	0.85
[It would be important to introduce sustainability practices also in procurement, selecting more sustainable products and suppliers]	4.13	0.89
[Greener surgical practices should be prioritized on the agenda of hospitals and other stakeholders (e.g., financing institutions and governments)]	4.10	0.95
[Greener surgical practices should be prioritized on the agenda of healthcare top managers]	4.09	0.96
[Greener surgical practices should be properly reported]	4.08	0.88
[Disposable items should be replaced by reusable items (e.g., drapes, metalware, …)]	4.01	1.02
[Green management should be a priority in all medical specialities.]	3.99	0.94
[Grants and awards should prioritize greener surgical practices]	3.98	0.97
[Greener surgical practices should be embedded into clinical guidelines]	3.98	0.99
[Greener surgical practices should be prioritized on the agenda of scientific societies]	3.95	1.00
[Ratios and indexes connected to surgical practices should be calculated to measure and report sustainability in the surgical department]	3.94	0.91
[Non-technical skills can lead to more sustainable surgical practices]	3.91	0.96
[Patients should be educated about environmental issues and outcomes connected with clinical activities and choices]	3.90	0.98
[Green management should be a priority in surgery]	3.88	1.01
[Hospitals and other stakeholders (e.g., financing institutions and governments) will appreciate greener surgical practices]	3.83	1.03
[Greener surgical practices should be preferred even if they are more expensive]	3.75	0.99
[Changing clinical practices can lead to fewer issues than traditional practices]	3.65	0.92
[Patients should be aware of the most sustainable clinical options when co-deciding with their physician]	3.63	1.05
[Patients will appreciate greener surgical practices]	3.56	1.11
[Sustainability issues are particularly relevant in my clinical area of activity]	3.49	1.18
[Greener management of surgical practice can lead to better clinical outcomes]	3.45	1.04
[Patients will have more trust in greener surgical practices]	3.38	1.06

### Sustainability habits in surgical practice

Interestingly, when it came to rating sustainability habits (most of which borrowed from the Royal Colleges’ checklist), the mean ranking is pretty low, with the best-rated item at 3.68 with a standard deviation of 1.27 and the lowest-rated factor at 2.56 with a standard deviation of 1.2.

More generally, most items show a standard deviation higher than 1, meaning that the sample is widely distributed among those who claim that several green practices are in place in their institutions and those who declare the contrary.

It should be noted that the home institution “promotes greener clinical practices in general terms”was among the worst rated of factors.

Table 4 reports the results related to questions about sustainability habits in surgical practice.

**Table 4:** Results—Sustainability habits in surgical practice

Think of your clinical practice. On a scale from 1 to 5, where 1 = strongly disagree and 5 = strongly agree, how would you rate the following statements?		
Please note that all the elements should be rated according to your knowledge		
	MEAN	SD
[My hospital/institution recommends ensuring only appropriate contents in sharps bins (sharps/drugs).]	3.68	1.27
[My hospital/institution recommends opening only those sets that are needed and, when they are needed, by integrating supplementary items into sets]	3.38	1.26
[My hospital/institution recommends that damaged reusable equipment is repaired, encouraging active maintenance.]	3.34	1.25
[My hospital/institution recommends avoiding clinically unnecessary interventions (e.g., antibiotics, catheterization, histological examinations).]	3.33	1.26
[My hospital/institution recommends arranging metals/battery collection where possible.]	3.27	1.30
[My hospital/institution recommends powering off lights, computers, ventilation, AGSS, and temperature control when the theatre is empty.]	3.14	1.33
[My hospital/institution recommends using non-infectious offensive waste (yellow/black tiger), unless clear risk of infection.]	3.10	1.28
[My hospital/institution installed several automatic or pedal-controlled water taps.]	3.05	1.47
[My hospital/institution recommends avoiding all unnecessary equipment (e.g., swabs, single-use gloves).]	2.99	1.28
[My hospital/institution recommends the creation of surgeon preference lists for each operation—separate essential vs. optional items to have ready on side.]	2.98	1.32
[I have a clear understanding and picture about greener clinical practices adopted by my hospital/institution.]	2.90	1.17
[My hospital/institution recommends opting for reusables, hybrid, or remanufactured equipment instead of single-use (e.g., diathermy, gallipots, kidney-dishes, light handles, quivers, staplers, energy devices)].	2.88	1.29
[My hospital/institution accurately measures operating room sterile supply waste.]	2.83	1.29
[My hospital/institution uses reusable textiles (including theatre hats, sterile gowns, patient drapes, and trolley covers).]	2.81	1.35
[My hospital/institution is supplied by firms adopting sustainable practices]	2.77	1.21
[My hospital/institution recommends using domestic or recycling waste streams for all packaging.]	2.71	1.27
[After the first water scrub of the day, I use alcohol rub for the subsequent cases.]	2.68	1.48
[My hospital/institution endorses the reduction in water and energy consumption.]	2.68	1.23
[My hospital/institution employs sustainable inputs (e.g., from renewable sources, and/or made of biodegradable materials, and/or coming from recycling processes)]	2.66	1.20
[My hospital/institution recommends avoiding the use of single-use surgical packs.]	2.61	1.29
[My hospital/institution promotes greener clinical practices in general terms.]	2.57	1.14
[My hospital/institution recommends switching to low-carbon alternatives (e.g., skin sutures vs. clips, loose prep in gallipots).]	2.57	1.28
[My hospital/institution recommends recycling or using lowest carbon-appropriate waste streams as appropriate.]	2.56	1.20

### Sustainability measures in hospitals and institutions

A list of items related to measures was provided, with participants needing to declare their presence or absence. The “I do not know” option was also possible. The practice with more consensus was about the regular maintenance of surgical equipment and the presence of automatic or pedal-controlled water taps.

Several management issues like procurement, equipment lease, or the type of energy sources in use received mainly “I do not know” responses. Multidisciplinary green teams or committees are reported as either present at only a few institutions or participants are not aware of their existence.

Table 5 highlights the results of such measures.

**Table 5:** Results—Sustainability measures in hospitals and institutions

My hospital/institution participates in one or more of the following sustainability measures				
Please note that all the elements should be rated according to your knowledge				
	YES	NO	I do not know	TOT
[Regular maintenance of surgical equipment/repair of damaged surgical equipment]	319	92	39	450
[Automatic or pedal-controlled water taps]	263	165	22	450
[Formal mechanism for reducing clinically unnecessary interventions (e.g., antibiotics, catheterisation, histological examinations)]	241	159	50	450
[Regular waste audits to ensure appropriate separation of sharps, biologic, non-infectious waste]	230	132	88	450
[Use of reusable, hybrid, and remanufactured equipment (e.g., diathermy, gallipot, kidney-dishes, light handles, quivers, staplers, energy devices)]	213	196	41	450
[Reusable textiles (gowns, drapes, etc.)]	204	222	24	450
[Limits single-use surgical packs]	200	215	35	450
[Battery/metal recycling program]	196	143	111	450
[Regular waste audits for accurate recording of used and wasted]	193	150	107	450
[Use of alcohol rub over water scrub]	191	216	43	450
[Participation in domestic or recycling waste streams]	177	161	112	450
[Automatic powering off of unused lights, computers, ventilation, AGSS, and temperature control when operating theatre is not in use]	171	215	64	450
[Standardized, consensus-based surgeon preference cards for each operation]	160	229	61	450
[Employs leased machines or IT instruments]	126	189	135	450
[Formal mechanism for incentivizing less wasteful, lower-carbon, or more environmentally sustainable practices (e.g., skin sutures vs. clips, loose prep in gallipots)]	114	256	80	450
[Employs shared machine or IT instruments with other institutions]	108	224	118	450
[Hospital electricity generated from sustainable energy source (wind, hydro, solar, etc.)]	101	240	109	450
[Purposefully contracts with organizations that adopt sustainable practices]	87	192	171	450
[Multidisciplinary Sustainability/Green Committee with meetings at regular intervals]	72	226	152	450

### Promotion of green/environmental sustainability practices

Participants were asked to give their opinions about possible tools to promote green practices, according to a knowledge translation theoretical lens[26].

Interestingly, participants backed the presence of multidisciplinary green teams[27], and the participation in specific training modules. Digital solutions were also highly rated, while visual instruments like billboards and leaflets were rated as low.

Table 6 reports the results of the possible promotional tools.

**Table 6:** Results—Promotion of green/environmental sustainability practices

Think of your clinical practice in your home hospital/institution. On a scale from 1 to 5, where 1 = strongly disagree and 5 = strongly agree, which of the following tools do you think SHOULD be used/encouraged to promote sustainability/greener clinical practices?		
	MEAN	SD
[Multidisciplinary Green Teams]	4.11	1.10
[Invitation to attend specific training courses]	4.08	1.09
[Digital solutions (e.g., APPs, sensors, smart grid, â€¦)]	4.06	1.08
[Invitation to report best practices at congresses or events]	3.99	1.08
[Websites]	3.94	1.18
[Illuminated signs and meters (e.g., reporting energy consumption or other data)]	3.88	1.13
[Newsletters]	3.63	1.29
[Corporate reports]	3.62	1.20
[Billboards]	3.41	1.32
[Leaflets]	3.12	1.36

### Aims and impacts of green/environmental sustainability practices

Last but not least, participants were asked to rate the perceived aims and impacts of green practices. In accordance with the results of the other sections, general attention was granted to the topic, underling the positive contribution to the natural environment. Notably, the hospital’s reputation was also highly valued. Patient’s engagement was, on the other hand, the worst-rated factor.

Table 7 reports the findings of the question related to the aims and impacts of environmental sustainability practices.

**Table 7:** Results—Aims and impacts of green/environmental sustainability practices

Accordingly, to your knowledge and/or perception, what is the tangible impact of greener clinical practice WITHIN your hospital / institution? On a scale from 1 to 5, where 1 = low, and 5 = remarkable, how would you rate the following statements?		
Please note that all the elements should be rated according to your knowledge		
	MEAN	SD
[Positive contribution for the environment]	4.00	1.13
[Reputation of the hospital / institution]	3.99	1.07
[My personal commitment]	3.97	1.04
[Cost reduction]	3.64	1.15
[Better quality of care]	3.58	1.19
[More engagement of the patient]	3.41	1.17

### Differences by groups

The analysis of participants' perceptions and behaviors regarding environmental Sustainability revealed varying trends based on Gender (Sex), Institution, and Position, even if not too relevant also when statistically significant.

Specifically, females showed slightly more familiarity with the concepts (72% vs. 68% for males), but this difference was not statistically significant (*p* = 0.4). Acceptance scores differed by gender, with females reporting higher scores (4.08 vs. 3.88 for males, *p* = 0.019). Non-academic institutions demonstrated lower promotion practices (3.60 vs. 3.85 for academic institutions, *p* = 0.008), while "Board-certified surgeons" reported lower promotion (3.51) and aims (3.57) scores. Notably, differences in acceptance scores between "Senior consultants" and "Residents" (*p* = 0.026) and in aims scores between academic and non-academic institutions (*p* = 0.012) were statistically significant. Other comparisons across categories did not yield significant differences (*p* > 0.05). These results shed light on nuanced patterns within these groups' perceptions of, and engagement with, sustainable healthcare practices, as reported in the following Table 8.

**Table 8:** Results–Differences by groups

	Sex			Institution			Position				
	Male, *N* = 356^a^	Female, *N* = 89^a^	*p* value^b^	Academic, *N* = 339^a^	Non-academic, *N* = 111^a^	*P* value^b^	Division chief or head, *N* = 74^a^	Senior or consultant, *N* = 180^a^	Board-certified surgeon, *N* = 123^a^	Resident, *N* = 73^a^	*P* value^c^
Familiarity	241 (68%)	64 (72%)	0.4	237 (70%)	69 (62%)	0.13	54 (73%)	129 (72%)	72 (59%)	51 (70%)	0.068
Acceptance	3.88 (0.72)	4.08 (0.57)	0.019	3.95 (0.69)	3.81 (0.74)	0.051	3.95 (0.74)	3.97 (0.67)	3.91 (0.76)	3.74 (0.63)	0.026
Habits	2.97 (0.90)	2.81 (0.76)	0.2	2.97 (0.90)	2.83 (0.82)	0.12	3.05 (0.95)	2.98 (0.87)	2.92 (0.90)	2.74 (0.78)	0.12
Measures	0.48 (0.29)	0.51 (0.30)	0.4	0.49 (0.30)	0.47 (0.29)	0.6	0.47 (0.28)	0.52 (0.29)	0.46 (0.32)	0.48 (0.29)	0.2
Promotion	3.80 (0.78)	3.76 (0.86)	> 0.9	3.85 (0.76)	3.60 (0.89)	0.008	4.02 (0.78)	3.83 (0.74)	3.74 (0.87)	3.51 (0.79)	< 0.001
Aims	3.79 (0.87)	3.72 (0.93)	0.6	3.83 (0.86)	3.57 (0.97)	0.012	3.99 (0.81)	3.99 (0.93)	3.71 (0.91)	3.57 (0.80)	0.013

^a^n (%); Mean (SD)

^b^Pearson’s Chi-squared test; Wilcoxon rank sum test

^c^Pearson’s Chi-squared test; Kruskal–Wallis rank sum test

## Discussion

As the healthcare industry significantly impacts the planet and its resources, environmental sustainability is becoming a hotly contested topic[18, 31]. Therefore, adopting sustainable practices must become a priority not only for healthcare policymakers and executives but also for clinicians, who must factor in sustainability when making medical decisions. Therefore, a cultural shift is necessary, as the environmental dimension of sustainability should be as essential as the social and economic ones when it comes to healthcare and surgery.

The purpose of the study was to use a survey to inquire surgeons about their perceptions about the topic and the current sustainability practices in place at their home institutions. Our initial pilot study yields intriguing results.

First of all, most participants were aware of some of the concepts related to green sustainability, even applied to surgery. Many recognized the connection with topics such as climate change, carbon footprint, waste management, use of energy and other resources. Still, a few were aware of the more general concept of providing good quality healthcare today without compromising the future generations’ possibility to do the same. Notably, several surgeons declared that sustainability was not their priority, as patients’ goals and outcomes should come first.

The patient’s sphere is particularly interesting as a result. Indeed, while some general concepts reach a broad consensus (for example, the need for Governments and institutions to back these “new” practices, even by offering funds and policies), the patient remains out of this scheme. Surgeons doubt that patients would appreciate (more) green practices than traditional ones or they would trust more environmentally sustainable habits or treatment options. This seems in contrast with other industries like, for instance, retail, where high degrees of corporate social responsibility and environmentally friendly initiatives are highly appreciated by customers and users and can represent a company or institution’s competitive advantage and branding [32–34]. Again, the perception is that when it comes to the “heart” and ultimate aim of healthcare, environmental sustainability is not the first priority but rather a welcomed bonus. Notably, panelists do believe that employing and disseminating greener practices can, indeed, improve the hospital’s image and reputation.

Coming to greener practices, a variety of ideas and opinions emerge. Indeed, there are no practices that reach broad consensus besides some, for instance, repairing surgical equipment, that represent organizational best practices even beyond their potential impact on sustainability. Notably, most participants did not agree with the fact that their home institution would second environmentally friendly practices in general terms, or at least, this message is not clear enough.

Several of the items recommended as best practices by the forerunners UK Royal Colleges were unknown by most panelists, like procurement strategies, equipment use contracts, and energy sources, meaning that hospital managers do not disclose their practices or, again, the message is not clear.

Still, our results show a general interest toward the topic of environmental sustainability applied to surgical and medical practices. Participants highly recognized the contribution to the environment and the potential role of healthcare policymakers, while still not encompassing these concepts in their daily routine and clinical decision-making, with a perceived low degree of acceptance. However, they seem open to dialogue by promoting, for instance, the establishment of multidisciplinary green teams [27] or committees with the aim of disseminating best practices and explaining concepts (like procurement or energy sources) that seem far from surgery, or participating in dedicated training courses. The potential role of technology remains an open question, with surgeons believing in its contribution to a greener surgical journey for patients. Although no huge differences emerge, some groups appear more sensitive or positive toward the topic, for instance, academic institutions that seem more open to innovation and cutting-edge issues, in line with previous WSES investigations [28].

Concerning the latest contribution of the UK Royal Colleges, it emerges how most practices are not in place, or surgeons are not fully aware of their existence.

All in all, the debate is now open, but the route seems still long. While surgeons believe in the need to preserve the natural environment and its resources, they may do so more as individuals rather than as professionals. Several doubts arise, and there seems to be a lack of guidance and translation, especially by hospitals and hospital managers. Institutions, governments, health agencies, and scientific societies should engage more in the dialogue by discussing, disclosing, promoting, and reporting their greener initiatives. Clinical studies about the feasibility of greener medical options should be encouraged. Dissemination and education devoted to patients and citizens as potential healthcare users should be provided, and their outcomes should be measured to understand the real impact.

## Limitations

Although the STAR initiative received a respectable response rate of 450 participants (approximately 50%), the sample is not geographically representative. Indeed, the majority of participants are employed in Europe, specifically in Italy. Some of our findings may have been influenced by the unique circumstances of the Italian and European contexts, including the type of healthcare service. Our limitations and the increasing interest in the topic may inspire new in-depth studies and investigations, including the description of single initiatives as recommended by the Royal Colleges [20].

## Conclusions

In concluding our work, we should recall the premise that inspired it. Environmental sustainability is a crucial topic in all industries, including healthcare. The future of environmental sustainability will need the engagement of physicians and surgeons, who will need to include it as one more guiding principle in their clinical decision-making.

Our results underline uncertainty and lack of knowledge about the topic and poor guidance by policymakers like governments, institutions, and health agencies. A paradigm shift is imperative to promote sustainable practices and translate clearly the negative impact of some daily routines or work habits on the planet. Acceptance and knowledge translation represent, therefore, two crucial concepts on the agenda of healthcare policymakers and leaders in the next few years to transform healthcare and surgery into (more) environmentally friendly fields, in accordance with the 2030 UN SDG Agenda [35].

While environmental sustainability must not change the ultimate aim of surgery and healthcare to serve patients, the sector and its leaders can do more to mitigate the field’s harmful impact on the planet. The cutting-edge work of the UK professional bodies should encourage more initiatives, including tests and trials, guidelines, dissemination activities through congresses, meetings and training, industry engagement, and cross-fertilization with other disciplines. It is a long but exciting journey, which should be on the agenda of multidisciplinary academic and practice leaders.

### Supplementary Information

**Additional file 1**. STAR Study Group members.

## Data Availability

The datasets used and/or analyzed during the current study are available from the corresponding author upon reasonable request.

## Appendix 1

**STAR Survey**

**Part 1–Descriptive statistics**

Omissis.

**Part 2–Perception of Green/environmental Sustainability**

**1. Are you familiar with the term green/environmental sustainability?**

***Yes/No***

1. Yes
2. No

**2 How would you describe green/environmental sustainability applied to surgery?**

***open question***

**Part 3–Sustainability acceptance and change management**

**3.** **Think of your clinical practice. On a scale from 1 to 5, where 1 = strongly disagree and 5 = strongly agree, how would you rate the following statements?**

***Likert scale 1 to 5***

1. Sustainability issues are particularly relevant in my clinical area of activity
2. Green management should be a priority in all the industrial sectors
3. Green management should be a priority in all medical specialities.
4. Green management should be a priority in surgery
5. Patients should be aware of the most sustainable clinical options when co-deciding with their physician
6. Patients will appreciate greener surgical practices
7. Hospitals and other stakeholders (e.g., financing institutions and governments) will appreciate greener surgical practices
8. Patients will have more trust in greener surgical practices
9. Disposable items should be replaced by reusable items (e.g., drapes, metalware, …)
10. Grants and awards should prioritize greener surgical practices
11. Greener surgical practices should be embedded into clinical guidelines
12. Greener surgical practices should be prioritized on the agenda of scientific societies

13. Greener surgical practices should be prioritized on the agenda of healthcare top managers

14. Greener surgical practices should be prioritized on the agenda of hospitals and other stakeholders (e.g., financing institutions and governments)
15. Greener surgical practices should be preferred even if they are more expensive
16. Technology can lead to more sustainable surgical practices
17. Surgical instruments should consider environmental efforts (eg. limiting the use of energy, non-recyclable materials and the creation of waste)
18. Non-technical skills can lead to more sustainable surgical practices
19. Greener surgical practices should be properly reported
20. Ratios and indexes connected to surgical practices should be calculated to measure and report sustainability in the surgical department
21. Changing clinical practices can lead to fewer issues than traditional practices
22. Greener management of surgical practice can lead to better clinical outcomes
23. Clinicians should be educated about environmental issues and outcomes connected with clinical activities and choices
24. Patients should be educated about environmental issues and outcomes connected with clinical activities and choices

25. It would be important to introduce sustainability practices also in procurement, selecting more sustainable products and suppliers
26. Governments should incentivize green surgical practices

**Part 4–Sustainability habits in surgical practice**

**4. Think of your clinical practice. On a scale from 1 to 5, where 1 = strongly disagree and 5 = strongly agree, how would you rate the following statements?**

**Please note that all the elements should be rated according to your knowledge**

***Likert scale 1 to 5 + NA***

1. I have a clear understanding and picture about greener clinical practices adopted by my hospital/institution.
2. My hospital/institution promotes greener clinical practices in general terms.
3. My hospital/institution uses reusable textiles (including theatre hats, sterile gowns, patient drapes, and trolley covers).
4. My hospital/institution endorses the reduction in water and energy consumption.
5. After the first water scrub of the day, I use alcohol rub for the subsequent cases.
6. My hospital/institution installed several automatic or pedal-controlled water taps.
7. My hospital/institution recommends avoiding clinically unnecessary interventions (e.g., antibiotics, catheterisation, histological examinations).
8. My hospital/institution recommends the creation of surgeon preference lists for each operation—separate essential vs. optional items to have ready on side.
9. My hospital/institution recommends avoiding the use of single-use surgical packs.
10. My hospital/institution recommends opening only those sets that are needed and, when they are needed, by integrating supplementary items into sets
11. My hospital/institution recommends avoiding all unnecessary equipment (eg swabs, single-use gloves).
12. My hospital/institution recommends opting for reusables, hybrid, or remanufactured equipment instead of single-use (e.g., diathermy, gallipots, kidney-dishes, light handles, quivers, staplers, energy devices).
13. My hospital/institution recommends switching to low-carbon alternatives (e.g., skin sutures vs. clips, loose prep in gallipots).
14. My hospital/institution recommends recycling or using lowest carbon-appropriate waste streams as appropriate.
15. My hospital/institution recommends using domestic or recycling waste streams for all packaging.
16. My hospital/institution recommends using non-infectious offensive waste (yellow/black tiger), unless clear risk of infection.
17. My hospital/institution recommends ensuring only appropriate contents in sharps bins (sharps/drugs).
18. My hospital/institution recommends arranging metals/battery collection where possible.
19. My hospital/institution recommends that damaged reusable equipment is repaired, encouraging active maintenance.
20. My hospital/institution recommends powering off lights, computers, ventilation, AGSS, and temperature control when the theatre is empty.

21. My hospital/institution is supplied by firms adopting sustainable practices
22. My hospital/institution employs sustainable “inputs” (e.g., from renewable sources, and/or made of biodegradable materials, and/or coming from recycling processes)
23. My hospital/institution accurately measures operating room sterile supply waste.

**5. My hospital/institution participates in one or more of the following sustainability measures**

**Please note that all the elements should be rated according to your knowledge**

***Yes, No, I do not know***

**Hospital Policies**

1. Multidisciplinary Sustainability/Green Committee with meetings at regular intervals
2. Standardized, consensus-based surgeon preference cards for each operation
3. Formal mechanism for reducing clinically unnecessary interventions (e.g., antibiotics, catheterisation, histological examinations)
4. Formal mechanism for incentivizing less wasteful, lower-carbon, or more environmentally sustainable practices (e.g., skin sutures vs. clips, loose prep in gallipots)
5. Hospital electricity generated from sustainable energy source (wind, hydro, solar, etc.)
6. Purposefully contracts with organizations that adopt sustainable practices
7. Automatic powering off of unused lights, computers, ventilation, AGSS, and temperature control when operating theatre is not in use
8. Employs leased machines or IT instruments
9. Employs shared machine or IT instruments with other institutions

Operative Supplies

10. Use of alcohol rub over water scrub
11. Automatic or pedal-controlled water taps
12. Limits single-use surgical packs
13. Reusable textiles (gowns, drapes, etc.)
14. Use of reusable, hybrid, and remanufactured equipment (e.g., diathermy, gallipot, kidney-dishes, light handles, quivers, staplers, energy devices)
15. Regular maintenance of surgical equipment/repair of damaged surgical equipment

Waste Management

16. Regular waste audits to ensure appropriate separation of sharps, biologic, non-infectious waste
17. Regular waste audits for accurate recording of used and wasted
18. Participation in domestic or recycling waste streams
19. Battery/metal recycling program

**5. Think of your clinical practice in your home hospital/institution. Which of the following tools are used to promote sustainability/greener clinical practices?**

***Tick all the ones that apply***

1. Billboards
2. Leaflets
3. Newsletters
4. Websites
5. Corporate reports
6. Invitation to attend specific training courses
7. Invitation to report best practices at congresses or events
8. Illuminated signs and meters (e.g., reporting energy consumption or other data)
9. Digital solutions (e.g., APPs, sensors, smart grid, …)
10. Other (please type) ____________
11. None of the above

**6. Think of your clinical practice in your home hospital/institution. Which of the following tools do you think SHOULD be used/encouraged to promote sustainability/greener clinical practices?**

***Likert scale 1 to 5 ***

1. Billboards
2. Leaflets

3. Newsletters
4. Websites
5. Corporate reports
6. Multidisciplinary Green Teams
7. Invitation to attend specific training courses
8. Invitation to report best practices at congresses or events
9. Illuminated signs and meters (e.g., reporting energy consumption or other data)
10. Digital solutions (e.g., APPs, sensors, smart grid, …)
11. Other (please type) ____________

**7. Accordingly to your knowledge and/or perception, what is the tangible impact of greener clinical practice WITHIN your hospital / institution? On a scale from 1 to 5, where 1 = low, and 5 = remarkable, how would you rate the following statements?**

**Please note that all the elements should be rated according to your knowledge**

***Likert scale 1 to 5 + NA***

1. Positive contribution for the environment
2. More engagement of the patient
3. Cost reduction
4. Better quality of care
5. My personal commitment
6. Reputation of the hospital / institution

## Abbreviations

WSES: World society of emergency surgery
CHERRIES: Checklist for reporting results of internet E-surveys
SD: Standard deviation
SGDs: Sustainable development goals
UN: United Nations
